# Calprotectin as a Potential Biomarker for Inflammation in Lung Cancer Patients

**DOI:** 10.3390/diagnostics16050780

**Published:** 2026-03-05

**Authors:** Selen Karaoğlanoğlu, Hüseyin Erdal, Müge Sönmez

**Affiliations:** 1 Department of Pulmonology, Faculty of Medicine, Ordu University, 52200 Ordu, Türkiye; 2 Department of Medical Genetics, Faculty of Medicine, Aksaray University, 68100 Aksaray, Türkiye; huseyinerdal@aksaray.edu.tr; 3 Department of Internal Medicine, Division of Medical Oncology, Faculty of Medicine, Ordu University, 52200 Ordu, Türkiye; mugesonmez@odu.edu.tr

**Keywords:** calprotectin, lung cancer, inflammation, biomarker

## Abstract

**Background/Objectives:** Calprotectin (CLP), a calcium-binding protein complex released predominantly from neutrophils and monocytes, plays a key role in the inflammatory response. Increased levels of CLP have been reported in various inflammatory and malignant conditions. This study aimed to evaluate serum CLP concentrations and their associations with hematological and biochemical parameters in patients with lung cancer. **Methods:** This prospective observational study included newly diagnosed lung cancer patients and a healthy control group. Demographic data, routine laboratory parameters, CLP levels, and inflammatory indices including neutrophil-to-lymphocyte ratio (NLR), platelet-to-lymphocyte ratio (PLR), systemic immune–inflammation index (SII), systemic inflammation response index (SIRI), and pan-immune–inflammation value (PIV) were recorded. Comparisons were made between groups and across tumor molecular profile, cancer stages, and metastasis status. Correlation and ROC analyses were performed. **Results**: Serum CLP levels were significantly higher in the lung cancer group compared with healthy controls (*p* < 0.001). Among molecular subgroups, patients with positive molecular testing had significantly elevated CLP levels compared with negative and untested groups (*p* = 0.025). CLP did not differ significantly across cancer stages or metastasis status (*p* > 0.05). CLP showed a positive correlation with the SIRI (*r* = 0.323; *p* = 0.004) and PIV (*r* = 0.395; *p* < 0.001). ROC analysis revealed that CLP demonstrated good diagnostic performance for lung cancer, with an AUC of 0.930 (95% CI: 0.849–0.976), sensitivity of 79.5%, and specificity of 92.3%. Among inflammatory indices, PIV (AUC = 0.863) and SIRI (AUC = 0.810) also showed high diagnostic accuracy. **Conclusions:** CLP levels are significantly elevated in lung cancer and show strong discriminative ability, outperforming commonly used inflammatory indices. Although CLP is not specific to lung cancer, it may serve as a supportive, noninvasive biomarker reflecting inflammatory burden when interpreted alongside clinical evaluation, imaging findings, and other laboratory parameters.

## 1. Introduction

Lung cancer remains one of the leading causes of cancer-related morbidity and mortality worldwide [1,2,3,4]. It is broadly classified into two major histological types, namely small-cell lung cancer (SCLC) and non-small-cell lung cancer (NSCLC), with the latter accounting for approximately 85% of all cases [5]. The primary etiological factor for lung cancer is cigarette smoking, which is responsible for nearly 80–90% of cases. Other risk factors include exposure to radon, asbestos, air pollution, and genetic susceptibility [6]. Despite improvements in diagnostic and therapeutic approaches, the prognosis of lung cancer remains poor, mainly due to late diagnosis at advanced stages [3].

In recent years, screening programs have been developed to facilitate early detection of lung cancer, particularly among high-risk populations. Low-dose computed tomography (LDCT) has been shown to reduce lung cancer mortality by enabling the identification of tumors at earlier, more treatable stages [7,8,9]. Nevertheless, current screening strategies are primarily based on LDCT imaging, and ongoing research continues to explore complementary approaches that may further improve risk stratification and early detection [7,10,11]. Furthermore, as smoking habits persist in many populations, screening programs face several drawbacks, including high false positive rates, overdiagnosis, radiation exposure, and high costs [12]. Consequently, there is an ongoing need for novel, noninvasive, and cost-effective biomarkers that can aid in early detection and provide insights into tumor biology and inflammation-related mechanisms.

Calprotectin is a well-established marker of innate immune activation and systemic inflammation and has been reported to be elevated in a variety of inflammatory conditions, including infections, autoimmune diseases, and several malignancies, and also in lung carcinogenesis by promoting DNA damage, angiogenesis, and immune evasion [13,14,15]. Among inflammation-related molecules, calprotectin, a heterodimer of S100A8 and S100A9 proteins, has emerged as a potential biomarker reflecting systemic inflammation and immune activation [16]. Calprotectin (CLP) is mainly released from neutrophils and monocytes during inflammatory responses and has been implicated in various inflammatory diseases [17,18,19,20,21]. Several studies have also investigated the diagnostic value of CLP in respiratory diseases, including pneumonia, respiratory failure, and lung cancer [22,23,24,25]. A review of the literature reveals that numerous biomarkers have been studied for their diagnostic potential in lung cancer, especially in NSCLC, which represents the predominant histological subtype [26].

Recent studies have suggested that increased CLP concentrations may also be associated with lung cancer, reflecting both tumor-associated inflammation and systemic immune responses [27]. However, the available data are still limited, and the relationship between CLP and routine hematological or biochemical inflammatory parameters has not yet been fully elucidated.

Therefore, the present study aimed to evaluate serum CLP levels in patients with lung cancer and investigate their relationship with hematological and biochemical parameters. We hypothesized that CLP might serve as a potential biomarker reflecting the inflammatory state associated with lung cancer.

## 2. Materials and Methods

This cross-sectional study was conducted at the Medical Oncology Unit of Ordu State Hospital, Türkiye, between April 2024 and December 2024. Patients with a newly confirmed diagnosis of lung cancer based on histopathological evaluation were considered for inclusion according to the predefined inclusion and exclusion criteria.

### 2.1. Inclusion and Exclusion Criteria

Eligibility criteria required patients to be 18 years of age or older, have a definitive histopathological diagnosis of lung cancer, have no prior history of chemotherapy, to not have used antioxidant therapy or herbal supplements at the time of participation or within the previous three months, to not have recently received medications such as steroids or N-acetylcysteine, and provide voluntary written informed consent. Patients were excluded if they had previously undergone chemotherapy; had used antibiotics, antioxidant therapy, or steroids in the recent period; or declined to participate. The control group consisted of healthy adults matched for age criteria, and the same exclusion rules were applied to ensure comparability.

The diagnosis of lung cancer was established histopathologically based on tissue biopsy specimens obtained through bronchoscopic biopsy, transthoracic needle biopsy, or surgical procedures.

Tumor staging was performed according to the International Association for the Study of Lung Cancer (IASLC) Tumor–Node–Metastasis (TNM) classification system, 8th edition [28], using clinical and radiological findings at the time of diagnosis.

Molecular analyses were performed on tumor tissue samples using immunohistochemistry (IHC), fluorescence in situ hybridization (FISH), polymerase chain reaction (PCR), and next-generation sequencing (NGS), in accordance with routine clinical practice and institutional protocols.

### 2.2. Data Collection and Laboratory Analysis

Peripheral venous blood samples were collected from both patients and controls after at least 8 h of fasting. Demographic characteristics and laboratory findings were obtained from electronic hospital records.

### 2.3. Hematological Analyses

Complete blood count parameters were analyzed using an automated hematology analyzer (XN-1000, Sysmex Europe GmbH, Norderstedt, Germany). This is a closed-system analyzer that performs measurements using fluorescence flow cytometry technology across all analytical modes. Measured hematological parameters included neutrophil, lymphocyte, monocyte, and platelet counts. Based on these values, inflammatory indices were calculated as follows: neutrophil-to-lymphocyte ratio (NLR = neutrophils/lymphocytes), platelet-to-lymphocyte ratio (PLR = platelets/lymphocytes), systemic immune inflammation index (SII = platelets × neutrophils/lymphocytes), systemic inflammation response index (SIRI = neutrophils × monocytes/lymphocytes), and pan immune–inflammation value (PIV = neutrophils × platelets × monocytes/lymphocytes).

### 2.4. Biochemical Analyses

For biochemical analyses, approximately 8 mL of blood was collected into serum separator tubes without anticoagulant. Samples were allowed to clot at room temperature for approximately 20 min and then centrifuged at 3000 rpm for 10 min. Serum levels of C-reactive protein (CRP), urea, creatinine, alanine aminotransferase (ALT), aspartate aminotransferase (AST), and lactate dehydrogenase (LDH) were measured using an automated biochemistry analyzer (Cobas 8000 serisi c702 modüler analizörü (Roche Diagnostics, GmbH, Manheim, Germany)). Serum CLP concentrations were quantified using an enzyme-linked immunosorbent assay (ELISA) method on the ELx-800 (BioTek^®^) analyzer with a commercial kit (Elabscience Human CLP ELISA Kit, Catalog No: E-EL-H2357, Houston, TX, USA). The results were expressed in ng/mL. This study was approved by the Ethics Committee of Ordu University (approval date: 28 April 2023; approval number: 113) and conducted in full accordance with the Declaration of Helsinki.

### 2.5. Statistical Analysis

Statistical analyses were carried out using MedCalc statistical software (version 20.009; Ostend, Belgium). The Shapiro–Wilk test was applied to assess the normality of data distribution. Categorical variables were expressed as frequencies and percentages, whereas continuous variables were presented as mean ± standard deviation (SD) or as median with 25th and 75th percentiles, depending on the distribution characteristics. For comparisons of laboratory and inflammatory parameters between study groups, the independent-samples *t*-test was used for normally distributed variables and the Mann–Whitney U test was used for non-normally distributed variables. In the tables, normally distributed data were reported as mean ± SD, while non-normally distributed data were displayed as median (25th–75th percentile). The Chi-square test was used to evaluate categorical variables.

To compare multiple independent groups, one-way ANOVA or the Kruskal–Wallis test was performed based on normality and homogeneity of variances. Post hoc analyses were conducted using the Tukey–Kramer test following one-way ANOVA and Dunn’s test following the Kruskal–Wallis test. Multiple-comparison tests were performed at a significance level of α = 0.05, and group differences were represented by letter superscripts in the tables.

The strength and direction of relationships between numerical variables were examined using correlation analysis. Pearson correlation was applied when normality assumptions were met, and Spearman correlation was used otherwise. Receiver operating characteristic (ROC) curve analysis was performed to assess and compare the diagnostic performance of inflammatory markers. The optimal cutoff values were determined using the Youden J index, and sensitivity, specificity, positive predictive value (PPV), negative predictive value (NPV), and area under the curve (AUC) with 95% confidence intervals (CIs) were reported. A *p*-value of <0.05 was considered statistically significant.

## 3. Results

The sociodemographic characteristics of the patients included in the study are presented in Table 1. There was no significant difference between the lung cancer and control groups in terms of age (66.0 [62.3–73.0] vs. 69.0 [63.3–72.0] years, *p* = 0.885) and gender distribution (*p* = 0.747). Among patients with lung cancer, adenocarcinoma was the most common tumor subtype (46.2%), followed by squamous cell carcinoma (41.0%) and small-cell lung cancer (12.8%). Nearly half of the patients were diagnosed as stage 4 (46.2%) at the time of diagnosis. Molecular testing was performed in 17 (43.6%) patients and was positive in 13 (33.3%) patients.

A detailed comparison of the laboratory parameters between the lung cancer and control groups is presented in Table 2. Patients with lung cancer exhibited significantly higher white blood cell (WBC) and neutrophil counts compared with the control group (*p* = 0.000 and *p* < 0.0001, respectively). Monocyte levels were also markedly elevated in the lung cancer group (*p* < 0.0001), whereas lymphocyte and platelet counts did not differ significantly between the groups (*p* = 0.260 and *p* = 0.112, respectively). The lung cancer group had higher mean platelet volume (MPV) values (*p* = 0.009) and lower hemoglobin levels (*p* = 0.010).

Regarding biochemical parameters, serum CLP levels were significantly elevated in lung cancer patients compared with the control group (*p* < 0.0001). Similarly, C-reactive protein (CRP), urea, and lactate dehydrogenase (LDH) concentrations were markedly higher in the lung cancer group (all *p* < 0.0001). Alanine aminotransferase (ALT) levels were significantly lower in the lung cancer group (*p* = 0.002), while aspartate aminotransferase (AST) values did not show a statistically significant difference between the two groups (*p* = 0.068).

A comparison of inflammatory indices between the two groups is summarized in Table 3. Although NLR and PLR values were numerically higher in the lung cancer group compared with the controls, the differences did not reach statistical significance. In contrast, SII levels were significantly elevated in lung cancer patients (*p* = 0.031). Furthermore, both the SIRI and PIV showed pronounced increases among patients with lung cancer compared with the control group (*p* < 0.0001 for both), indicating a substantial inflammatory burden associated with malignancy.

A comparative analysis of CLP and inflammatory indices across the tumor subtypes is summarized in Table 4. Although serum CLP levels tended to be higher in patients with adenocarcinoma compared with those with squamous cell carcinoma and SCLC, the difference did not reach statistical significance (*p* = 0.115). Similarly, no significant differences were observed among the subtypes regarding NLR, PLR, SII, SIRI, and PIVs (all *p* > 0.05). Notably, patients with SCLC exhibited numerically higher values for inflammatory markers including NLR, PLR, SII, SIRI, and PIV compared with the other subgroups; however, these elevations did not attain statistical significance, likely due to the limited sample size in this subgroup.

A statistically significant difference in serum CLP levels was observed among patients according to molecular testing status (*p* = 0.025). The median serum CLP levels varied across the molecular-positive, molecular-negative, and unexamined groups, with median CLP levels of 2.26, 1.88, and 1.80, respectively, as summarized in Table 5. In contrast, no statistically significant differences were observed among the groups for NLR, PLR, SII, SIRI, and PIV (all *p* > 0.05). These findings suggest that CLP levels may be associated with molecular positivity, whereas other inflammatory indices did not vary according to molecular testing status.

The distribution of CLP and inflammatory parameters across different cancer stages is summarized in Table 6. Although CLP levels tended to increase from stage 1 to stage 4, the differences among stages did not reach statistical significance (*p* = 0.323). Similarly, no significant variations were observed for NLR, PLR, SII, SIRI, and PIVs across the cancer stages (all *p* > 0.05). These results suggest that while there is a numerical trend toward higher inflammatory burden at advanced stages, the differences were not statistically significant in this cohort.

Correlation analysis between laboratory parameters and inflammatory indices is presented in Table 7. As expected, WBC and neutrophil counts showed positive correlations with NLR, SII, SIRI, and PIV (all *p* < 0.05), while lymphocyte counts were negatively correlated with these indices (all *p* < 0.05), reflecting their direct involvement in the calculation of these inflammatory markers. Monocyte counts demonstrated strong positive correlations with the SIRI and PIV (*r* = 0.600 and 0.720, respectively; both *p* < 0.0001).

In contrast, the correlations between biochemical parameters and inflammatory indices were considered more clinically meaningful. Hemoglobin levels were inversely correlated with NLR, SII, SIRI, and PIV (*p* ≤ 0.002). Serum calprotectin (CLP) showed significant positive correlations with the SIRI (*r* = 0.323, *p* = 0.004) and PIV (*r* = 0.395, *p* < 0.001), indicating an association with systemic inflammatory burden. Similarly, C-reactive protein (CRP) was positively correlated with the SIRI and PIV (*p* ≤ 0.009). Urea and lactate dehydrogenase (LDH) also exhibited significant positive correlations with the SIRI and PIV (*p* < 0.05).

The diagnostic performance of CLP and other inflammatory parameters for lung cancer was evaluated using ROC curve analysis (Table 8). CLP demonstrated good diagnostic accuracy with an AUC of 0.930 (95% CI: 0.849–0.976), sensitivity of 79.5%, specificity of 92.3%, PPV of 91.2%, and NPV of 81.8% at a cutoff value of >1.45 ng/mL (*p* < 0.0001). Among the inflammatory indices, PIV and SIRI showed good discriminative performance, with AUCs of 0.863 and 0.810, respectively (*p* < 0.0001 for both). NLR, PLR, and SII had lower diagnostic accuracy (AUCs of 0.620, 0.551, and 0.642, respectively), with only the SII reaching statistical significance (*p* = 0.026).

**Figure 1:**
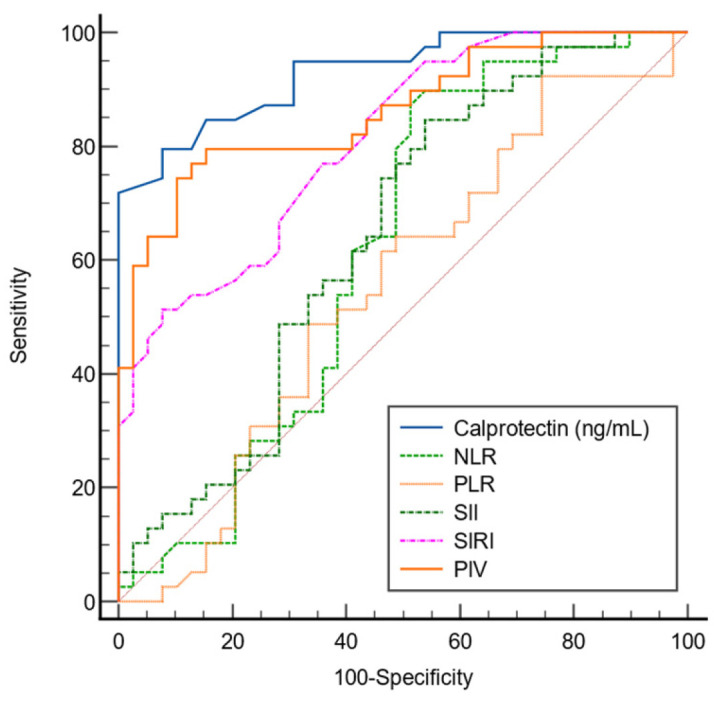
ROC curve analysis comparing calprotectin and inflammatory parameters (NLR, PLR, SII, SIRI, PIV) for the diagnosis of lung cancer. Calprotectin demonstrated the highest AUC (0.930), followed by PIV (0.863) and SIRI (0.810), while NLR, PLR, and SII showed lower diagnostic performance.

The ROC curve analysis comparing the diagnostic performance of CLP and inflammatory parameters is illustrated in Figure 1. CLP demonstrated the highest diagnostic accuracy for lung cancer, with an AUC of 0.930, surpassing all other inflammatory indices. PIV and SIRI also showed good discriminatory ability, with AUCs of 0.863 and 0.810, respectively. In contrast, NLR, PLR, and SII had lower AUC values, indicating limited diagnostic performance.

## 4. Discussion

This study evaluated serum calprotectin levels and other systemic inflammatory indices and explored their associations in patients with lung cancer. Our findings demonstrated that CLP levels were significantly elevated in lung cancer patients compared with healthy controls and were closely associated with other markers of systemic inflammation, such as the SIRI and PIV. Importantly, CLP showed good diagnostic performance for lung cancer, outperforming conventional inflammatory indices in terms of specificity and overall accuracy. Although trends were observed with tumor stage, metastasis status, and molecular testing, statistical significance was primarily observed for CLP in relation to molecular testing positivity. Given that the majority of patients included in this study had locally advanced or advanced-stage lung cancer, serum calprotectin (CLP) should not be interpreted as a marker for early detection. Instead, our findings suggest that CLP may reflect systemic inflammatory burden and disease-related inflammatory activity in lung cancer patients, particularly at advanced stages.

In a previous study investigating six cancer-associated serum molecules in 72 patients with lung cancer and 56 controls, CLP emerged as one of the strongest discriminators among the evaluated biomarkers, showing high diagnostic accuracy and demonstrating a sensitivity of 83% and specificity of 87% when combined with EGF and sCD26 in a three-marker panel [27]. The authors suggested that CLP may serve as a valuable noninvasive tool for distinguishing lung cancer patients from individuals with benign pulmonary conditions or healthy subjects. In our study, CLP alone demonstrated a remarkably high AUC value of 0.930, along with sensitivity of 79.5% and specificity of 92.3%. This concordance reinforces the notion that systemic inflammatory activity, reflected by increased CLP release, is an important pathophysiological feature of lung cancer.

There is evidence in the literature that S100A8/A9 expression is associated with specific NSCLC subtypes and with more advanced disease stages, and several studies have reported increased S100A8/A9 levels particularly in advanced or metastatic cases, linking this elevation to poorer prognosis [29]. In the present study, serum CLP levels showed a gradual increase from stage 1 to stage 4 disease, as well as numerically higher values in patients with metastatic involvement compared with those without metastasis. However, these trends did not reach statistical significance. The stepwise rise in CLP concentrations across advancing stages and in the presence of metastasis may nonetheless reflect an association between systemic inflammatory burden and tumor progression. The lack of statistical significance is likely attributable to the limited sample size within stage and metastasis-defined subgroups, which may have reduced the power to detect modest but clinically relevant differences. Therefore, our findings should be interpreted as hypothesis-generating and warrant confirmation in larger, prospectively designed cohorts with sufficient representation of each disease stage and metastatic status [30].

Our findings are consistent with previous studies highlighting the diagnostic utility of serum CLP in lung cancer. In the study by Blanco-Prieto et al. [27], CLP was identified as one of the strongest discriminatory markers within a multi-analyte panel, and our results similarly demonstrated excellent diagnostic performance, with a high AUC and specificity that surpassed conventional inflammatory indices. These data suggest that CLP may serve as a clinically useful triage or complementary test for distinguishing lung cancer from healthy individuals, particularly in symptomatic populations. Given its strong discriminative capacity, elevated CLP levels may also enhance diagnostic suspicion when integrated with imaging modalities, such as in the assessment of indeterminate pulmonary nodules. However, although promising, our single-center and cross-sectional data are insufficient to support its use as a screening strategy at this stage. From a clinical perspective, the diagnostic value of serum calprotectin may be enhanced when used in combination with clinical risk factors (such as smoking history), radiological findings, and other laboratory parameters. Its noninvasive nature and relatively high diagnostic accuracy suggest that calprotectin could serve as a supportive and noninvasive biomarker in lung cancer suspicion or risk stratification, rather than as a standalone diagnostic test. Finally, given that elevated calprotectin levels may also be observed in non-malignant inflammatory conditions, careful patient selection and exclusion of active infections or inflammatory diseases are essential when interpreting serum calprotectin levels.

This study has several limitations that should be considered when interpreting the findings. First, the single-center design and relatively limited sample size may restrict the generalizability of the results to broader populations. Second, the cross-sectional nature of the study does not allow for causal inferences between serum calprotectin levels and lung cancer development or progression. Third, calprotectin is a nonspecific inflammatory marker and may be influenced by concomitant inflammatory conditions, infections, or comorbidities that could not be completely excluded, despite careful patient selection. Additionally, serial measurements of calprotectin were not available; therefore, changes in calprotectin levels over the course of the disease or in response to treatment could not be evaluated. Lung cancer comprises heterogeneous histopathological subtypes with distinct inflammatory microenvironments, which may influence circulating biomarker levels. Calprotectin is predominantly released from neutrophils and monocytes and has been associated with tumor-related inflammation, particularly in smoking-related lung cancers such as squamous cell carcinoma. In contrast, adenocarcinoma may exhibit different inflammatory profiles. Therefore, the diagnostic performance of serum calprotectin may vary according to tumor cell type. However, the relatively small sample size in each histological subgroup in our study limited robust cell-type-specific analyses and may have reduced the statistical power to detect subtype-dependent differences. Larger studies stratified by histopathological subtype are needed to clarify the relationship between lung cancer cell type and calprotectin-based diagnostic accuracy. Finally although smoking history is an important factor when evaluating calprotectin levels, detailed smoking exposure data were not available for all participants. Therefore, subgroup analyses based on smoking exposure could not be performed. Prospective, multicenter studies with external validation are required to establish its generalizability, and longitudinal designs assessing serial CLP measurements will be essential to clarify its prognostic value in relation to treatment response and survival. In addition, emerging approaches integrating radiomics with blood-based multiomics biomarkers may improve early lung cancer detection and risk stratification. Such multimodal models could enhance generalizability and support clinical decision-making beyond single-biomarker strategies, as suggested in recent radiomics research [31].

## 5. Conclusions

In conclusion, this study demonstrates that serum calprotectin levels are significantly elevated in patients with lung cancer and are associated with established systemic inflammatory indices. These findings suggest that serum CLP reflects the inflammatory burden accompanying lung cancer rather than serving as a disease-specific biomarker.

Given its lack of specificity and its known elevation in other inflammatory and malignant conditions, serum CLP should not be considered a standalone diagnostic marker for lung cancer. However, as a noninvasive and easily measurable parameter, CLP may have potential value as a supportive biomarker when interpreted in conjunction with clinical assessment, imaging findings, and other laboratory markers of inflammation.

Further large-scale, prospective studies with well-defined control groups, balanced disease stages, and inclusion of other cancer types and inflammatory comorbidities are required to clarify the diagnostic and prognostic utility of serum calprotectin and to establish clinically meaningful cutoff values for its potential use in lung cancer management.

## Figures and Tables

**Table 1:** Demographic features of lung cancer and control groups.

		Groups				*p*-Value
		Lung Cancer		Control		
		*N* = 39		*N* = 39		
		Median	25P–75P	Median	25P–75P	
Age (years)		66.0	62.3–73	69.0	63.3–72	0.885
		*n*	%	*n*	%	
Gender	Female	5	12.8	6	15.4	0.747
	Male	34	87.2	33	84.6	
Tumor subtype	Adenocarcinoma	18	46.2			
	Small-Cell Lung Cancer	5	12.8			
	Squamous	16	41			
Cancer stage	Stage 1	5	12.8			
	Stage 2	6	15.4			
	Stage 3	10	25.6			
	Stage 4	18	46.2			
Molecular testing	Negative	4	10.3			
	Not examined	22	56.4			
	Positive	13	33.3			

**Table 2:** Comparison results on laboratory parameters in the lung cancer and control groups.

	Groups				*p*-Value
	Lung Cancer		Control		
	*N* = 39		*N* = 39		
Hematological results					
WBC (10^3^/L)	10.3	(7.7–12)	7.6	(5.3–9.4)	<0.001 **
Neutrophil (10^3^/L)	6.5	(4.7–8.4)	3.6	(2.8–5)	<0.0001 **
Lymphocyte (10^3^/L)	2.0	(1.33–3)	1.8	(1–2.7)	0.260
Monocyte (10^3^/L)	0.70	(0.6–0.98)	0.30	(0.2–0.5)	<0.0001 **
Platelet (10^3^/L)	296	(233.8–353.5)	262	(165–333.8)	0.112
MPV (fL)	9.7	0.86	9.1	0.98	0.009 *
Hemoglobin (g/dL)	13.0	(11.6–13.9)	13.5	(12.6–15.3)	0.010 **
Biochemical results					
Calprotectin (ng/mL)	2.05	(1.64–2.29)	1.12	(0.93–1.29)	<0.0001 **
CRP (mg/L)	10.0	(4.8–20.3)	3.0	(2–4)	<0.0001 **
Urea (mg/dL)	31.0	(23–38.8)	14.0	(12–17)	<0.0001 **
ALT (IU/L)	17.0	(14–23)	25.0	(18.3–30)	0.002 **
AST (IU/L)	20.0	(13.3–27)	16.0	(12–23.5)	0.068
LDH (IU/L)	228	(187.8–256.5)	167	(148.3–189)	<0.0001 **

* Significant difference at <0.05 level according independent *t*-test; means and standard deviations (SDs) are presented. ** Significant difference at <0.05 level according to Mann–Whitney U test; medians are presented and 25P–75P are shown in parentheses.

**Table 3:** Comparison of inflammatory parameters between the lung cancer and control groups.

Inflammatory Parameters	Groups				*p*-Value
	Lung Cancer		Control		
	*N* = 39		*N* = 39		
NLR	2.63	(2.03–5.35)	1.94	(1.41–4.59)	0.069
PLR	119.6	(100.4–207.1)	159.5	(105.6–374.9)	0.435
SII	899.2	(612.7–1401.7)	594.5	(314.1–1408.475)	0.031 **
SIRI	2.60	(1.4–4.28)	0.90	(0.33–1.78)	<0.0001 **
PIV	734.6	(451.2–1135.6)	237.6	(98.2–392.3)	<0.0001 **

Means and standard deviations (SDs) are presented. ** Significant difference at <0.05 level according to Mann–Whitney U test; medians are presented and 25P–75P are shown in parentheses.

**Table 4:** Comparison of CLP and inflammatory parameters among tumor subtypes.

	Tumor Subtype									*p*-Value
	Adenocarcinoma			Small-Cell Lung Cancer			Squamous			
	*N* = 18			*N* = 5			*N* = 16			
	Median	25P	75P	Median	25P	75P	Median	25P	75P	
Calprotectin (ng/mL)	2.20	1.75	2.40	1.80	1.56	2.19	1.85	1.35	2.09	0.115
NLR	2.54	2.03	4.50	6.62	4.62	9.00	2.56	2.03	3.48	0.226
PLR	137.3	103.7	186.3	208.5	167.4	359.1	109.8	99.7	178.4	0.075
SII	716.2	510.8	987.5	1500.9	995.3	2011.1	929.2	609.0	1043.5	0.193
SIRI	2.00	1.10	4.30	6.40	2.90	7.23	2.95	1.30	3.70	0.157
PIV	556.9	284.6	1080.0	900.6	647.4	1825.2	859.0	455.0	1130.3	0.451

**Table 5:** Comparison of CLP and inflammatory parameters according to molecular testing.

	Molecular Testing									*p*-Value
	Negative			Not Examined			Positive			
	*N* = 4			*N* = 22			*N* = 13			
	Median	25P	75P	Median	25P	75P	Median	25P	75P	
Calprotectin (ng/mL)	1.88 ^a^	1.35	2.05	1.80 ^ab^	1.40	2.15	2.26 ^ac^	2.05	2.45	0.025 **
NLR	2.39	1.89	7.37	2.47	1.94	5.54	3.20	2.47	5.09	0.432
PLR	106.8	93.5	148.8	122.3	100.0	226.4	147.5	113.2	223.5	0.463
SII	807.6	477.3	1034.6	929.2	642.7	1500.9	794.5	579.7	2176.0	0.857
SIRI	2.35	1.15	5.25	2.45	1.40	4.00	2.60	1.40	5.75	0.844
PIV	558.7	303.7	910.3	904.7	455.0	1119.6	663.0	391.7	2006.6	0.733

**: Kruskal–Wallis test. Superscript letters indicate the results of post hoc pairwise comparisons based on median values. Groups that do not share a common letter are statistically significantly different from each other (*p* < 0.05).

**Table 6:** Comparison of CLP and inflammatory parameters according to cancer stages.

	Lung Cancer Stages												*p*-Value
	Stage 1			Stage 2			Stage 3			Stage 4			
	*N* = 5			*N* = 6			*N* = 10			*N* = 18			
	Median	25P	75P	Median	25P	75P	Median	25P	75P	Median	25P	75P	
Calprotectin (ng/mL)	1.50	1.38	2.24	1.88	1.25	2.12	1.85	1.70	2.05	2.20	1.75	2.40	0.323
NLR	1.94	1.70	2.31	2.64	1.54	4.78	2.41	2.03	5.54	3.34	2.45	6.00	0.121
PLR	127.1	106.2	173.0	144.0	91.6	226.4	107.1	100.0	208.5	138.9	113.5	203.0	0.749
SII	864.6	452.1	1015.4	777.7	331.7	1901.5	807.6	650.0	1500.9	912.3	669.3	1938.0	0.805
SIRI	1.50	0.90	2.43	3.40	0.80	4.30	2.25	1.40	3.30	2.75	1.50	6.80	0.543
PIV	734.6	258.2	1039.6	948.1	195.2	1140.9	677.8	455.0	1158.2	662.7	462.7	1119.6	0.949

**Table 7:** Correlation analysis between laboratory parameters and inflammatory indices.

		NLR	PLR	SII	SIRI	PIV
		*N* = 78				
WBC (10^3^/L)	*r*	0.239	−0.166	0.343	0.452	0.555
	*p*-Value	0.035 **	0.146	0.002 **	<0.0001 **	<0.0001 **
Neutrophil (10^3^/L)	*r*	0.582	0.111	0.661	0.746	0.813
	*p*-Value	<0.0001 **	0.332	<0.0001 **	<0.0001 **	<0.0001 **
Lymphocyte (10^3^/L)	*r*	−0.767	−0.822	−0.627	−0.468	−0.268
	*p*-Value	<0.0001 **	<0.0001 **	<0.0001 **	<0.0001 **	0.018 **
Monocyte (10^3^/L)	*r*	0.123	−0.171	0.196	0.600	0.720
	*p*-Value	0.284	0.135	0.086	<0.0001 **	<0.0001 **
Platelet (10^3^/L)	*r*	−0.201	0.072	0.118	−0.047	0.264
	*p*-Value	0.078	0.529	0.305	0.683	0.020 **
MPV (fL)	*r*	0.237	0.087	0.181	0.235	0.179
	*p*-Value	0.036 **	0.451	0.112	0.038 **	0.116
Hemoglobin (g/dL)	*r*	−0.408	−0.221	−0.348	−0.445	−0.381
	*p*-Value	0.000 **	0.052	0.002 **	<0.0001 **	0.001 **
Calprotectin (ng/mL)	*r*	0.160	−0.069	0.172	0.323	0.395
	*p*-Value	0.163	0.547	0.132	0.004 **	0.000 **
CRP (mg/L)	*r*	0.010	−0.223	0.086	0.293	0.411
	*p*-Value	0.931	0.050	0.452	0.009 **	0.000 **
Urea (mg/dL)	*r*	0.190	−0.029	0.263	0.500	0.594
	*p*-Value	0.096	0.801	0.020 **	<0.0001 **	<0.0001 **
ALT (IU/L)	*r*	−0.106	−0.018	−0.174	−0.266	−0.354
	*p*-Value	0.356	0.874	0.127	0.018 **	0.002 **
AST (IU/L)	*r*	−0.048	−0.193	−0.166	−0.053	−0.105
	*p*-Value	0.676	0.090	0.148	0.647	0.362
LDH (IU/L)	*r*	0.120	−0.080	0.153	0.282	0.372
	*p*-Value	0.297	0.487	0.180	0.012 **	0.001 **

** Significant correlation coefficient at <0.05 level according to Spearman’s rank correlation.

**Table 8:** Diagnostic performance of calprotectin and inflammatory parameters for lung cancer.

	Cutoff	Sensitivity	Specificity	PPV	NPV	AUC (95% CI)		*p*-Value
Calprotectin (ng/mL)	>1.45	79.5	92.3	91.2	81.8	0.930	(0.849–0.976)	<0.0001
NLR	>1.72	89.7	46.2	62.5	81.8	0.620	(0.503–0.727)	0.072
PLR	≤358.9	92.3	25.6	55.4	76.9	0.551	(0.434–0.664)	0.442
SII	>480.7	84.6	46.2	61.1	75.0	0.642	(0.526–0.748)	0.026
SIRI	>2.5	51.3	92.3	87.0	65.5	0.810	(0.705–0.890)	<0.0001
PIV	>427.3	79.5	84.6	83.8	80.5	0.863	(0.767–0.931)	<0.0001

AUC: area under curve; CI: confidence interval.

## Data Availability

The data supporting the findings of this study are available from the corresponding author upon reasonable request. The data are not publicly available due to privacy and ethical reasons.

## Abbreviations

The following abbreviations are used in this manuscript:

SCLC	Small-cell lung cancer
NSCLC	Non-small-cell lung cancer
LDCT	Low-dose computerized tomography
CLP	Calprotectin
NLR	Neutrophil-to-lymphocyte ratio
PLR	Platelet-to-lymphocyte ratio
SII	Systemic immune inflammation index
SIRI	Systemic inflammation response index
PIV	Pan-immune–inflammation value
ELISA	Enzyme-linked immunosorbent assay
SD	Standard deviation
WBC	White blood cell
MPV	Mean platelet volume
CRP	C-reactive protein
LDH	Lactate dehydrogenase
ALT	Alanine aminotransferase
AST	Aspartate aminotransferase
